# Impacts of vitamin A deficiency on biological rhythms: Insights from the literature

**DOI:** 10.3389/fnut.2022.886244

**Published:** 2022-11-18

**Authors:** Xiangrong Guo, Hui Wang, Jian Xu, Hui Hua

**Affiliations:** ^1^Shanghai Key Laboratory of Embryo Original Diseases, The International Peace Maternity and Child Health Hospital, Shanghai Jiao Tong University School of Medicine, Shanghai, China; ^2^MOE-Shanghai Key Lab of Children's Environmental Health, Xinhua Hospital, Shanghai Jiao Tong University School of Medicine, Shanghai, China

**Keywords:** vitamin A, biological rhythm, retinoic acid, retinoic acid nuclear receptor, brain function

## Abstract

Vitamin A is essential for brain function, in addition to its important roles in vision, immunity, and reproduction. Previous studies have shown that retinoic acid (RA), the bioactive form of vitamin A, is involved in the regulation of various intracellular responses related to biological rhythms. RA is reported to affect the circadian rhythm by binding to RA receptors, such as receptors in the circadian feedback loops in the mammalian suprachiasmatic nucleus. However, evidence of the impacts of vitamin A deficiency (VAD) on biological rhythms is limited, and most of the related studies were conducted on animals. In this review, we described the physiological functions of biological rhythms and physiological pathways/molecular mechanisms regulating the biological rhythms. We then discussed the current understanding of the associations of VAD with biological rhythm disorders/diseases (sleep disorders, impairments in learning/memory, emotional disorders, and other immune or metabolism diseases) and summarized the currently proposed mechanisms (mainly by retinoid nuclear receptors and related proteins) for the associations. This review may help recognize the role of VAD in biological rhythm disorders and stimulate clinical or epidemiological studies to confirm the findings of related animal studies.

## Vitamin A derivatives and signaling

**Figure 1:**
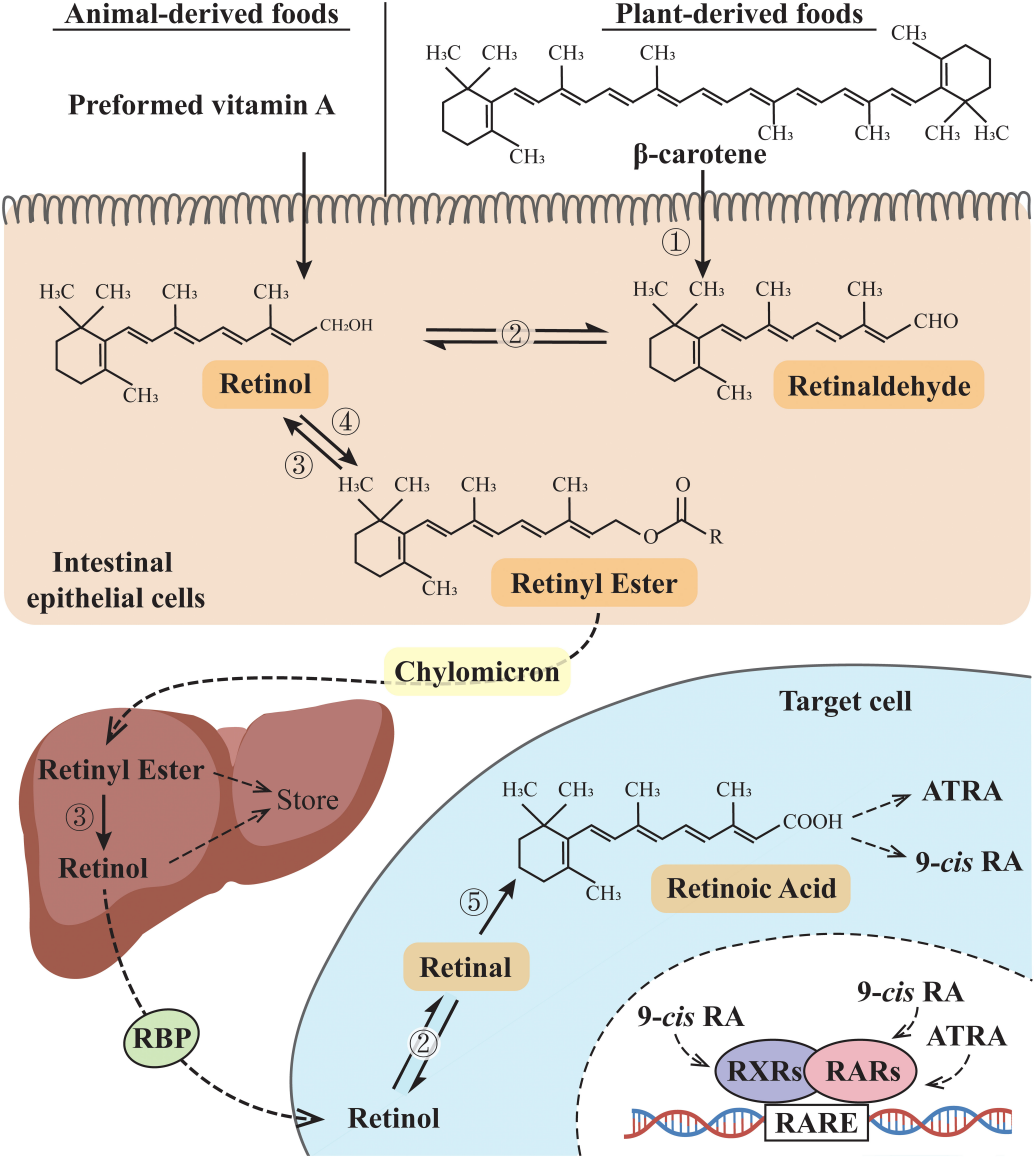
Vitamin A metabolic pathway. ➀ β-Carotene 15,15'-dioxygenases; ➁ Retinol dehydrogenases (RDHs): RDH1, RDH10, and short-chain dehydrogenase/reductase (SDR family) member 9 (DHRS9) (reversible reaction); ➂ retinyl esterase; ➃ lecithin: retinol acyltransferase (LRAT) or acyl-CoA: retinol acyltransferase (ARAT); ➄ retinaldehyde dehydrogenases (RALDH). RBP, retinol-binding protein; 9-*cis* RA: 9-*cis* retinoic acid; ATRA, all-*trans* retinoic acid; RXRs, retinoid X receptors; RARs, retinoic acid receptors; RARE, retinoic acid response elements.

Vitamin A is the first discovered fat-soluble vitamin and is primarily found in animal products or converted from dietary carotenoids in plant products. It is not a single compound but a group of derivatives including retinol, retinal, retinoic acid (RA), and some carotenoids according to different terminal functional groups. In general, vitamin A refers to retinol, while retinoid refers to a general term that includes vitamin A metabolites and compounds and exhibits vitamin A-like biological activity (1). Preformed vitamin A (usually from animal products) and provitamin A (including beta-carotene, usually from plant-derived food) are the two forms of vitamin A in the human diet. After being absorbed in the intestines, these two forms of vitamin A are converted to retinol and then oxidized to form retinal and RA to support the biological functions of vitamin A. The retinyl ester is the storage form of vitamin A in the liver and must be converted to retinol before being utilized, and these vitamin A derivatives are finally metabolized by CYP26 family enzymes (2, 3) (Figure 1). RA has several stereo-isomeric forms, including all-*trans* RA (ATRA), 13*-cis* RA, and 9-*cis* RA, and isomerization occurs under certain conditions (4). Together with its derivatives, vitamin A is involved in regulating diverse life activities, including cell proliferation and differentiation, vision, reproduction, embryogenesis, and immune function (5). Retinoic acid receptors (RARs) and retinoid X receptors (RXRs) are the two families of retinoid nuclear receptors (RNRs), and each family has three identified isotypes, namely, α, β, and γ (6). ATRA binds only to RARs, while 9-*cis* RA is the main ligand for RXRs and also has a binding affinity for RARs [Until recently, 9-*cis* RA is reported to be only detected in the pancreas (7, 8)]. RAR-RXR heterodimers form a functional structure that allows them to regulate gene expression. By binding to specific retinoic acid response elements (RAREs), RAR-RXR heterodimers can regulate downstream gene expression (9). When RA binds to RARs, the RAR-RXR dimers release the co-repressor proteins and activate downstream gene expression (10). However, when RA signals are not present, RAR/RXR dimers bind to co-repressors and recruit inhibitors to suppress the expression of downstream genes. Retinoid-related orphan receptors (RORs) belong to the ROR subfamily. Similar to RARs and RXRs in the gene sequence, RORs also have three isotypes, namely, α, β, and γ (11), and the endogenous ligands for RORs have not yet been confirmed. In addition to ATRA, substances confirmed to bind RORs include steroids, terpenoids, polyketides, and cardiac glycosides (12, 13). Since involved in related physiological functions, RORs may be potential targets in the treatment of biological rhythm disorders and related metabolic diseases (11, 14, 15).

Although vitamin A is present in a variety of foods, vitamin A deficiency (VAD) is still a problem worthy of attention in underdeveloped countries and regions in the world (16, 17). VAD is associated with an abnormal RA signaling pathway (18–20); for instance, the expression of RAR and RXR proteins decreased in rats or mice fed with a VAD diet (21–23). Animal studies also showed that prenatal VAD led to decreased RARα and RARβ expression, while vitamin A supplementation induced increased expression of RARβ (24). However, another animal study reported that in the hippocampus of VAD rats, the RARβ expression decreased, while the RARα expression increased (25).

## Physiological functions of biological rhythms

Biological rhythms regulate various physiological, biochemical, and behavioral processes in living organisms. As an invisible “timer”, biological rhythms can influence daily behavioral or physiological cycles, such as the sleep–wake cycle, body temperature, and blood pressure. The circadian rhythm, which is the most well-known biological rhythm, has been widely studied.

In mammals, the circadian system can be divided into the central clock and the peripheral clock. The central clock is controlled by the suprachiasmatic nucleus (SCN) in the anterior part of the hypothalamus directly above the optic chiasm bilateral to the third ventricle (26), producing a central rhythm. The spatiotemporal single-cell analysis revealed eight major cell types (each with a specific pattern of circadian gene expression) and five subtypes of SCN neurons (each with specific combinations of molecules) in the mouse SCN, displaying different circadian rhythmicity and light responsiveness (27). Intercellular coupling mediated by neuropeptides helps produce and maintain synchronous oscillation (28). The peripheral clock is composed of peripheral oscillators located in various peripheral tissues. Interestingly, the peripheral circadian clock contains similar molecular regulation mechanisms as the central circadian clock and is the basis for the normal functioning of various peripheral tissues (29). The central rhythm from the central clock can drive or synchronize the peripheral rhythm through neuro-humoral pathways, while the peripheral rhythm can, in turn, affect and regulate the central rhythm through periodic activities such as daily diet and sleep (30). In addition, the peripheral rhythm retains a certain degree of autonomy (31). As a pacemaker of central rhythm, the SCN can keep its own rhythm. Besides, the SCN can also make responses and changes under the influence of the external environment, such as light, food intake, and social activities, so that individuals can adapt to the external environment (32–34). Furthermore, SCN neurons synthesize GABA, vasopressin, vasoactive intestinal peptides, and other signaling molecules, which directly or indirectly regulate the circadian release of hormones, including melatonin and corticotropin-releasing factor (CRF), to bring the central rhythm information to peripheral tissues. This provides the possibility for the association between biological rhythm dysfunction and related disorders/diseases (35, 36). The coordination and interaction between the central and peripheral rhythms are vital for maintaining normal physiological functions. Disturbances in the central or peripheral rhythm can lead to a wide range of diseases (37, 38).

## Roles of vitamin A in regulating biological rhythms

### Relationships between vitamin A and biological rhythms

The associations between vitamin A and biological rhythms were shown to occur in different organisms such as insects, birds, and mammals (citations in Table 1). Vitamin A may affect biological rhythms, including the central rhythm produced by the SCN and peripheral cell rhythms. Evidence of the effects of VAD on biological rhythms is limited, and all related studies used nonhuman animals. A recent study on monarch butterflies induced CRISPR/CRISPR-associated protein 9-mediated targeted mutagenesis and found that vitamin A in the brain, rather than in the compound eye, functioned in photoperiod responsiveness and could link the photoperiod cycle with a seasonal response (39). Therefore, the impact on the central rhythm has become a key issue when exploring the effect of vitamin A on biological rhythms. Sherman et al. reported that with the supplementation of ATRA, the amplitude and time phase of the rhythmic gene expression in mouse liver cells were significantly changed (40), suggesting a role of vitamin A signaling in peripheral rhythms. However, Shirai et al. reported that the expression patterns of clock genes in liver cells of VAD mice remained unchanged, indicating that vitamin A may not be essential for maintaining the peripheral rhythm in mammals (41).

**Table 1:** Animal studies on the association of vitamin A deficiency with biological rhythm dysfunctions.

**Author**	**Year**	**Country**	**Animal classification**	**Measures**	**Main findings**
**Rhythm dysfunction in the hypothalamus**					
Shearer et al. (110)	2010	England	CD-1 mice, Fischer 344 rats	The level of RA in hypothalamus	The prolonged photoperiod regulated RA related expression of synthetic enzymes and receptors which led to enhancement of RA signal.
Helfer et al. (129)	2012	England	Fischer 344 rats	RA activity levels in the hypothalamus	RA activity levels in the hypothalamus of photoperiod-sensitive Fischer-344 rats were reduced in the short-day condition. These lower RA activity levels could be explained by decreased expression of a whole network of RA signaling genes in the ependymal cells around the third ventricle and in the arcuate nucleus of the hypothalamus.
**Rhythm dysfunction in the hippocampus**					
Golini et al. (86)	2012	Argentina	Holtzman rats	RARα and RXRβ expression levels and the daily expression patterns of clock BMAL1, PER1, RORα and REVERB genes	Vitamin A deficiency reduced the level of RXRβ mRNA, changed the rhythm amplitude of the PER1, REV-ERB genes and REV-ERB protein, and phase-shifted the daily peaks of RORα protein as well as BMAL1, RORα, RC3 and BDNF mRNA levels.
Navigatore-Fonzo et al. (51)	2013	Argentina	Holtzman rats	The expression of Bmal1, Per1 and retinoic acid receptors (RARs, RXRβ) genes	Vitamin A deficiency may affect the circadian expression in the hippocampus by modifying the rhythmic profiles of retinoic acid receptors.
Navigatore-Fonzo et al. (84)	2014	Argentina	Holtzman rats	Hippocampal clock genes expression	Clock gene expressions affected by Vitamin A deficiency.
**Rhythm dysfunction in other brain tissues (such as the pineal gland)**					
Veerman et al. (130)	1983	Holland	Phytoseiid mite	Photoperiodic induction of diapause	Carotenoids and vitamin A restored the photoperiodic reaction in an eyeless predacious mite.
Iiams et al. (39)	2019	USA	The monarch butterfly	Key circadian clock genes	The vitamin A in the insect brain, which was photoperiod/clock-regulated, mediated the seasonal responses.
Chernysheva et al. (131)	2012	Russia	Wistar rats	The levels of PER1 protein	Retinol supplementation increased the expression of PER1 protein.
Fu et al. (132)	1998	Japan	Japanese Quail	Synthesis of melatonin in pineal gland	Vitamin A deficiency decreased the response of pineal gland to light and the variation of melatonin in Japanese Quail, and vitamin A supplement could restore their photoreactivity.
**Circadian rhythm of peripheral tissues/cells**					
Shirai et al. (41)	2006	Japan	Jcl: ICR mice	Expression of clock genes in the mouse liver	Vitamin A deficiency had no effects on the circadian expression of liver clock genes in mice. Dietary vitamin A was not essential for maintaining peripheral rhythms in mammals.
Sherman et al. (40)	2012	Israel	C57B1/6 mice	The expression level of clock genes in the serum, liver and jejunum	ATRA could induce significant changes in the amplitude and phase of the expression of rhythms genes in the mice liver cells.
Shirai et al. (49)	2006	Japan	NIH3T3 cells	Expression of clock genes in the NIH3T3 cells	RA bidirectionally regulated mPER1 gene expression in a bidirectional and E-box dependent manner.

Although only animal studies have directly focused on the effects of VAD on biological rhythms, human studies have also explored the possibility of the correlation between vitamin A or carotenoids and biological rhythmicity or chronotype (but not directly on the effects of VAD, as shown in Table 2). Previous human studies reported that lower vitamin A intake levels were associated with disturbed wake–sleep cycles (42), and crocetin (a natural carotenoid compound) supplementation improved sleep time and quality (43–46). However, Asane et al. did not find differences in serum vitamin A levels between the groups with and without insomnia symptoms in adolescent girls (47). Therefore, more human studies are needed to confirm the relationship between VAD and biological rhythm disorders.

**Table 2:** Findings of epidemiological studies related to the associations of vitamin A or carotenoid with biological rhythms.

**Author**	**Year**	**Country**	**Study type**	**Sample size**	**Main findings**
**Vitamin A**					
Sato-Mito et al. (42)	2011	Japan	Cross-sectional study	3,304	The midpoint of sleep was calculated as the midpoint between bedtime and wake-up time, and lower vitamin A levels were associated with a later midpoint of sleep. In addition, people with a later midpoint of sleep tend to eat irregularly.
Gromadzińska et al. (133)	2012	Poland	Cross-sectional study	708	In the postmenopausal women, the plasma vitamin A levels were significantly lower in night-shifts workers compared with day-workers.
**Carotenoid**					
Kuratsune et al. (43)	2010	Japan	Crossover Randomized Controlled Trial	21	Crocetin is a type of carotenoid compound, and the number of wakening episodes decreased after taking crocetin.
Beydoun et al. (134)	2014	U.S.A.	Cross-sectional study	4,979	Short sleep time is associated with decreased serum levels of total carotenoids.
Umigai et al. (44)	2018	Japan	Crossover Randomized Controlled Trial	30	Delta activity during deep sleep determined sleep quality. Compared with the control group, delta power significantly increased in the group of taking crocetin (an active constituent of saffron).
Lopresti et al. (45)	2020	Australia	Randomized Controlled Trial	placebo (*n =* 30), saffron (*n =* 33)	The group that consumed saffron reported reduced insomnia severity as well as the number of wakes after falling asleep. Saffron also improved alertness upon awakening and sleep quality scores.
Pachikian et al. (46)	2021	Belgium	Randomized Controlled Trial	placebo (*n =* 32), saffron (*n =* 34)	Saffron extract can improve sleep time and make it easier to fall asleep, and it is also beneficial to the quality-of-life parameters.

### Molecular mechanisms underlying the associations of VAD with biorhythm disorders

Although the exact mechanisms regulating biological rhythms among different species are different, there are some common features.

#### Dysfunctions of the transcription–translation feedback loops (TTFLs, the molecular basis of biological rhythm regulation) induced by VAD

**Figure 2:**
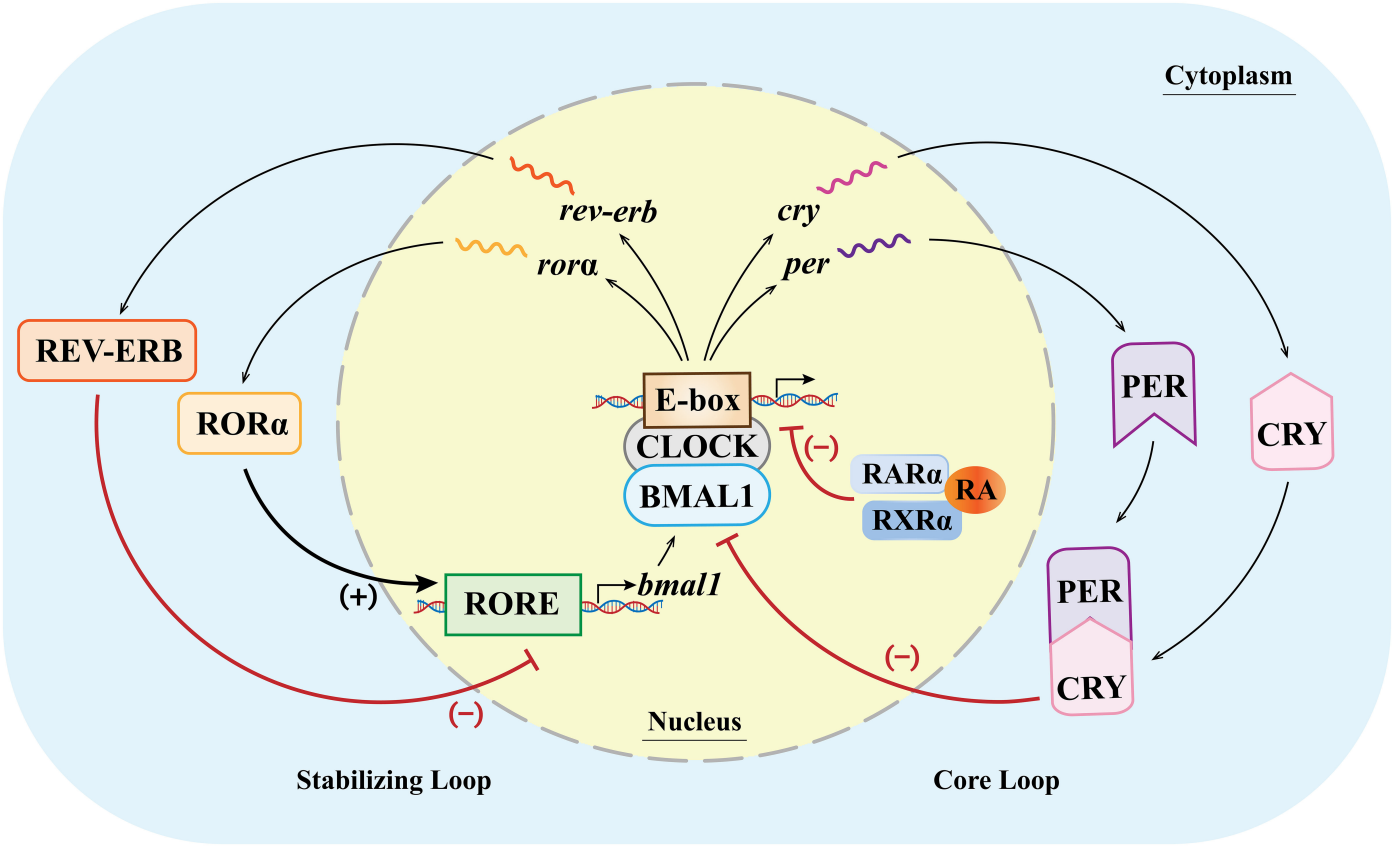
Clock genes and their transcription–translational feedback loops (TTFLs) at the cellular level (inside/outside the nucleus) in mammals. In the core loop, CLOCK and BMAL1 dimers bind to the E-box, promoting the expression of the Per and Cry genes and, in turn, inhibiting the CLOCK/BMAL1-dimer-dependent gene expression. In the stabilizing loop, the combination of the CLOCK/BMAL1 dimer and the E-box can activate the expression of RORα and REV-ERB. The accumulation of the REV-ERB molecule can inhibit the expression of the BMAL1 gene, while the accumulated RORα molecule can activate the expression of the BMAL1 gene (lower case italic characters and single wavy lines indicate mRNA). (+)/ → means activating gene expression; (–)/T means inhibiting gene expression.

Various clock genes and TTFLs constitute the molecular basis of biological rhythm regulation in the SCN (48). In mammals, the main molecules involved in the TTFLs include heterodimeric circadian locomotor output cycles kaput (CLOCK), brain and muscle Arnt-like protein 1 (BMAL1), periods (PERs 1, 2, 3), cryptochrome (CRYs 1, 2), RORs (α, β, γ), reverse erythroblastosis virus (REV-ERBs α/β), and RARα/RXRα (40, 49–51). The binding of the E-box and CLOCK/BMAL1 dimers (1) can activate the gene expression of PERs and CRYs, and then the molecules of PERs and CRYs form the PER/CRY complex and inhibit the CLOCK/BMAL1 dimer-dependent gene expression. The feedback loop in which the genes of PERs and CRYs are involved is called the core loop and (2) can also activate the expression of RORs and orphan nuclear receptor REV-ERBs (52–54). The feedback loop in which RORs and REV-ERBs are involved is called the stabilizing loop. In the stabilizing loop, the accumulation of REV-ERBs and RORs has different effects on the expression of the BMAL1 gene, and the accumulation speeds of the REV-ERBs and RORs are also different. Specifically, the accumulation of REV-ERBs is much faster than that of the RORs (55). The RORs and REV-ERBs competitively bind to the ROR response element (RORE) in the upstream promoter region of the BMAL1 gene (11), and the accumulation of REV-ERB molecules can inhibit the expression of the BMAL1 gene, while the accumulated ROR molecules can activate the expression of the BMAL1 gene (49, 50, 56). With the combined effects of these molecules, the expression of the genes related to biological rhythms is therefore regulated at multiple levels (Figure 2).

Because of multiple factors in the core and stabilizing loops, the duration of periodic change is about 24 h, forming an endogenously driven oscillating biological rhythm (circadian rhythm). Recent studies have shown that numerous cell types in the body can express clock genes (57). For example, the rhythms of smooth muscle cells can be affected by the changes in the PER2 expression induced by RA (58). Therefore, the SCN acts as the primary circadian oscillator, influencing and regulating the rhythms of peripheral cells, and coordinating the central and peripheral rhythms (59).

Although the role of vitamin A in regulating biological rhythms remains unclear, some studies have found that VAD or vitamin A supplementation after deficiency may affect the expression of various biological clock genes through nuclear RA receptors and proteins, which may be the potential mechanisms underlying the effects of VAD on the biological rhythms. Navigatore-Fonzo et al. showed that VAD might affect the transcription and post-transcription processes by affecting the transcriptional regulation of CAT and GPx mediated by RARs or RXRs, or by affecting the formation and functions of the BMAL1/CLOCK heterodimer (60). Animal experiments showed that with or without the presence of RARα, ATRA could significantly downregulate or upregulate the expression of the mouse PER1 gene and other circadian rhythm genes in an E-box-dependent manner (49). The binding of ATRA/9-*cis* RA to RXRα/RARα prevents the BMAL1/CLOCK heterodimer from activating the transcription of PER2 (58). These studies suggested that vitamin A and its derivatives can bind to RARs and RXRs to inhibit E-box-dependent clock gene expression mediated by the BMAL1/CLOCK dimer, which is the core circadian rhythm regulation pathway in the SCN.

Retinoid-related orphan receptors play an important role in the regulation of the SCN rhythm and are closely related to the RA signaling pathway. Both RORα and RORβ showed a rhythmic expression pattern in the SCN, while RORγ was not detected in the SCN (61). Mice lacking RORα or RORβ showed an abnormal circadian rhythm, while mice lacking RORγ did not show an abnormal rhythm (56, 62, 63). Nevertheless, RORγ has been found in several peripheral organs, such as the liver, and it may play a role in the rhythm of peripheral tissues (64). Stehlin-Gaon et al. found that under certain circumstances, ATRA might reversibly bind to RORβ and activate the related gene expression (65), but whether this finding remained under other circumstances was queried by other studies (66). Considering the important roles of RORα and RORβ in the feedback loops of circadian rhythms and the extensive participation of RORα and RORs in various life activities, it may be favorable to consider the impacts of RORs in the relationship between vitamin A, circadian rhythm, and related diseases/disorders.

#### Changes in the light signal reception induced by VAD and related circadian rhythm molecules

Vitamin A is essential for the normal physiological functioning of the eyes, and VAD may affect biological rhythms by affecting the reception of light signals. The light cycle is the primary driver that affects biological rhythms (32, 67). The light signals received by the retina are transmitted to the SCN through a process of photoentrainment, whereby light adjusts the central rhythm produced by the SCN to adapt to the external light–dark cycle. The photoentrainment involves a type of intrinsically photosensitive retinal ganglion cells (ipRGCs), whose axons can be sent to the SCN, making the ipRGC a potential photoreceptor for synchronizing biological clock to the environmental day–light cycle (68–70). The retinal and RA are the two main active vitamin A metabolites in the human body. In addition to RA, the retinal formation will also be impaired in the case of VAD. 11-*cis* retinal is the basic structure of visual pigments, oxidized 11-*cis* retinal combines with opsin to form rhodopsin in rod cells (71). Evidence indicates that ipRGCs detect light with a vitamin A-based photopigment, melanopsin. Melanopsin is a protein homologous to rhodopsin, using 11-*cis* retinal as the light-absorbing chromophore. In the vision process, under the light condition, 11-*cis* retinal is isomerized into all-*trans* retinal and dissociates with opsin, then reduction of all-*trans* retinal to all-*trans* retinol occurs, and all-*trans* retinol is subsequently transported to the retinal pigment epithelium (RPE). In the RPE, RPE65 protein helps convert all-*trans* retinal to 11-*cis* retinal *via* the enzymatic steps (72). In RPE65-knockout mice, the sensitivity of the ipRGCs is significantly decreased, while the supplementation of exogenous 9-*cis* retinal, 11-*cis* retinal, and all-*trans* retinal can restore the photosensitivity of ipRGCs, indicating that the photosensitivity of the ipRGCs requires vitamin A-based chromophores (69, 73). In the absence of light, oxidized 11-*cis* retinal is generated again and quickly interacts with opsin to form rhodopsin. Thus, protein conformational changes in a light–dark cycle can convert light signals into electrical signals and transmit the signals to the brain (74). Therefore, retinal may be reduced and even lost in the light–dark cycle. However, the human body cannot synthesize vitamin A by itself, so the supplementation of exogenous vitamin A is needed to ensure the normal circulation process. Therefore, vision loss or night blindness is a significant symptom in VAD cases (75), and a further lack of vitamin A may disturb the entrainment of biological rhythms. In addition, because retinal and RA have different sites of action in the body, their effects on biological rhythms are different, but associations between their effects still remain unknown. In VAD cases, a lack of retinal affects the normal light reception and light entrainment process in the eye, thus affecting biological rhythms, while a lack of RA affects the expression levels of rhythm-related molecules in the SCN and related peripheral tissue cells, leading to rhythm-related disorders. Although targeting different tissues or organs, they seem to exert a comprehensive effect on biological rhythm dysfunctions under VAD.

In zebrafish, interphotoreceptor retinoid-binding protein (IRBP) mediates the transport of retinoids in photoreceptors and RPE cells (76), and IRBP is expressed periodically, making IRBP an intermediary molecule in the effects of vitamin A on biological rhythms (77). In addition, the light cycle is easily associated with the circadian rhythm of melatonin secretion in the pineal gland. The light signals received by the SCN are converted into hormone signals in the pineal gland to regulate the rhythms of the whole body (78). Vitamin A is essential for the normal functioning of the pineal gland, and the circadian synthesis of RA and its signal transduction components occur in the pineal gland (79).

#### Changes in circadian rhythm molecules in the hippocampus and pineal gland induced by VAD

The pineal gland is a special organ in the association of vitamin A with biological rhythms. In the pineal gland, RA synthesis and RA signal-related genes show circadian changes (80). The pineal gland contains high levels of retinol and retinol-binding protein (81). Animal studies have demonstrated that vitamin A and its analogs may have potential genomic and non-genomic effects on biological rhythm regulation in the pineal gland. The transcription of the arylalkylamine N-acetyltransferase (AANAT), encoding the rhythm-generating melatonin synthetic enzyme, was upregulated under long-term RA intervention (79), and the peaks of the rhythms of the AANAT activity and melatonin level in VAD rats decreased significantly at night (82). RA can rapidly downregulate the phosphorylation of ERK 1/2 in the pineal gland of rats, to quickly regulate the expression of related genes and adapt to the real-time changing biological rhythms *via* non-genomic effects (79). Vitamin A is necessary for the process of the transmission of rhythmic signals from the SCN to the pineal gland and induces melatonin secretion (83), and the disturbance in this process may be one of the mechanisms underlying the rhythm dysfunction induced by VAD.

**Figure 3:**
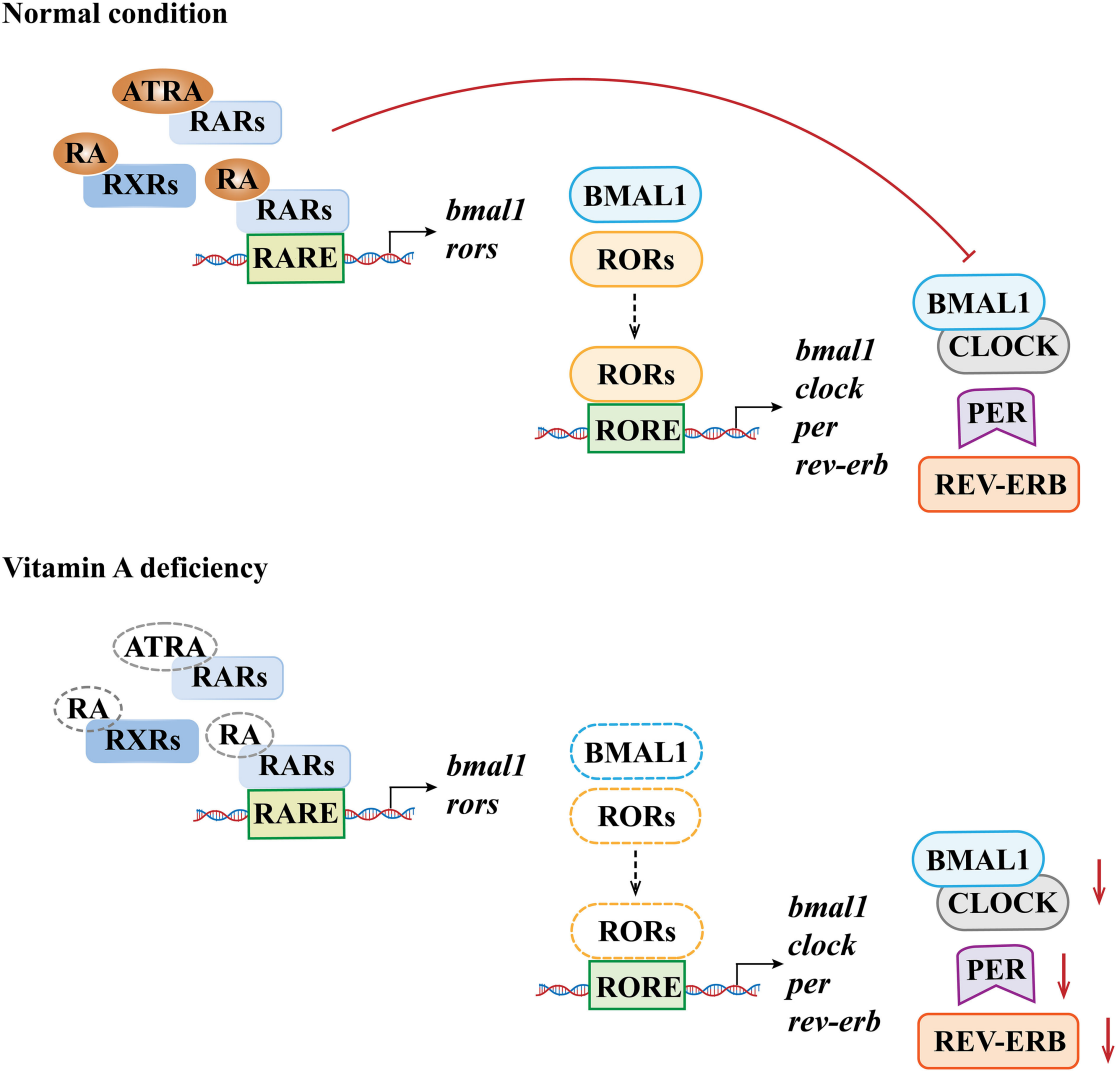
Molecular mechanisms underlying the effects of vitamin A deficiency on biological rhythm molecules. The dotted line denotes the lack of the susbstance.

As a peripheral rhythm oscillator, the hippocampus has its own rhythm while receiving signals of the central rhythm regulation from the SCN. Animal experiments confirmed that dietary VAD could affect the circadian rhythm and the expression of endogenous rhythm genes related to exercise in the rat hippocampus, changing the circadian rhythm of RAR expression, thereby altering the circadian gene expression rhythms of PER2, CRY1, and CRY2 (84). Another study showed that the circadian expression rhythm patterns of RORα and REV-ERB in the hippocampus of VAD rats changed (85), indicating that vitamin A concentrations had effects on the expression levels of RORα and REV-ERBα in the hippocampus. The underlying mechanism may be that RARE has been found in the regulatory regions of the BMAL1 and RORα, and a decrease in RARs and RXRs caused by VAD may lead to decreased signals acting on RARE; therefore, decreased expression of BMAL1 and RORα may then be induced. In addition, the regulatory regions of BMAL1, CLOCK, PER1, and REV-ERB genes have the RORE element, so the decreased RORα expression caused by VAD may further reduce the expression levels of these genes and ultimately affect the circadian rhythm in the hippocampus (11, 86) (Figure 3).

## Associations of VAD with biorhythm dysfunction-related diseases

### VAD and sleep disorders

It is well known that biorhythm disorders may present with an abnormal sleep cycle, and evidence suggests that VAD may adversely affect sleep, especially slow wave sleep (SWS) related to photoperiod. The SWS is of great significance for learning, memory, body growth, and repair (87). Under normal circumstances, delta oscillations (0.5–4 Hz) are common in a state of deep sleep and are representative brain waves during the SWS, while theta waves (4–7 Hz) occur in the transition between wake and sleep or lighter stages of sleep. Mice of some strains with fragmented sleep had reduced delta activity and increased theta activity during the SWS. The theta/delta (θ/δ) ratio is used to better quantify the altered EEG pattern. Changes in the relative contribution of the delta rhythm to the SWS or the θ/δ ratio were found to be associated with the changes in the expression levels of RARβ (88). Significant impairments in delta oscillations were also observed in VAD mice (89). Although these studies suggested the potential association between VAD and sleep disorders, the use of vitamin A and its bioactive derivatives in the treatment of sleep disorders caused by biological rhythm dysfunctions warrants future studies.

### VAD and learning/memory impairments

Both the rhythm-related genes (BMAL1, PERs, CRYs, and REV-ERBα) and RA receptors (RARα, RARβ, and RARγ) in the hippocampus have endogenous circadian rhythms (51, 60), which is critical for maintaining normal physiological functions of learning and memory (90, 91).

Retinoic acid plays a critical role in regulating neurogenesis, neuronal survival, and synaptic plasticity (92, 93). VAD may cause impairments in synaptic plasticity, learning, and memory in rats (94). Previous studies have demonstrated that RARα antagonists could damage long-term potentiation, indicating that RARα receptors may be involved in synaptic plasticity and the process of learning and memory (95). Prenatal marginal VAD was found to induce decreased RARα mRNA levels in the postnatal hippocampus (96, 97). Brain-derived neurotrophic factor (BDNF) and neurogranin (RC3) are key molecules involved in learning and memory, and BDNF and RC3 genes have rhythmic expression patterns in the hippocampus. A previous study showed that the VAD phase shifted the daily peaks of RC3 and BDNF mRNA levels in the rat hippocampus, affected their daily expression patterns, and might therefore affect the daily cognitive performances in rats (86, 98). Some interesting findings have been reported on crocetin. Crocetin is a natural carotenoid compound (not provitamin A carotenoid) and cannot be converted into vitamin A. However, studies have demonstrated that crocetin can promote the synaptic growth of hippocampal neurons to promote neural plasticity and neuronal communication (99), and plays roles in regulating learning, memory, and sleep. As a kind of carotenoid with a structure similar to that of carotene (the precursor of vitamin A), crocetin and its functions are mentioned here to raise consideration or inspire researchers to explore more possible functions of vitamin A in the future (100).

### VAD and emotional disorders/neuroendocrine disorders

The hypothalamus may play an important role in the association of VAD with biological rhythm-related emotional/neuroendocrine disorders. The hypothalamic-pituitary-adrenal (HPA) axis, which is composed of the paraventricular nucleus (PVN) of the hypothalamus, the anterior pituitary gland, and adrenal glands, controls reactions to stress and plays a prominent role in regulating mood and emotional statuses (101). CRF is a hypothalamic hormone and a core driver of the HPA axis (102). With extensive regulatory effects, RA signals can modulate the synthesis of CRF in the hypothalamus, and abnormal CRF signaling induced by VAD may affect the response of the body to stress, which may be associated with the development of anxiety and depression (103, 104). In addition, as a neuropeptide mainly synthesized by the PVN and supraoptic nucleus of the hypothalamus, oxytocin was found to play a role in modulating fear and stress responses (105, 106). RARE has been found in the regulatory region of the oxytocin gene (107); therefore, the decrease in RA signals caused by VAD may lead to decreased oxytocin synthesis, which may have potential associations with the development of anxiety or depression (108). In addition, thyroid hormone and RA had similar synthetic pathways and targets, and VAD may affect the pituitary thyroid axis (109, 110). Specifically, vitamin A is critical for iodine metabolism, and the 9-*cis* retinoic acid receptor (RXR) is a common heterodimerization partner for the thyroid hormone receptor (T3R). Therefore, VAD may lead to increased thyroid hormone synthesis and thyroid enlargement and aggravate the thyroid dysfunction caused by iodine deficiency (111, 112). These findings suggested a potential association between VAD and emotional/neuroendocrine disorders. However, although both the HPA activity and the oxytocin level in the brain have their own diurnal rhythms (113, 114), circadian rhythm disorders may induce emotional symptoms (115–117), and some clinically approved drugs that are used to treat depression and anxiety were found to play a role in regulating the biological clock (116, 118). Future studies are still warranted to confirm whether biological rhythm dysfunctions play a role in the association of VAD and emotional disorders.

### VAD and metabolism/immune dysfunctions/cancers

In addition to regulating the expression of biorhythm genes, RORs also play a crucial role in cell metabolism (lipid and steroid), proliferation, apoptosis, migration, immunity, and many other physiological or pathological processes (119–121). Melatonin, which is a hormone closely related to biological rhythms and mediated by RORα, regulates macrophage polarization, thereby preventing the transformation of atherosclerosis into cardiovascular and cerebrovascular events (122). Moreover, RORα may inhibit the effects of melatonin on testosterone synthesis in mice (123) and inhibit the proliferation, invasion, and migration of liver cancer cells by downregulating the chemokines (124). Since RORs constitute an important part of the molecular mechanism of biorhythm regulation, it can be speculated that RORs may be an intermediate step linking vitamin A, biological rhythms, and various diseases, including but not limited to metabolic syndromes, cardiovascular diseases, cancers, and inflammations (125, 126). Therefore, RORs may be a promising target for the treatment of immune, metabolic, and other biorhythm-related diseases (127, 128).

## Conclusion

In this review, we summarized the associations of vitamin A with biological rhythms and the potential impacts of VAD on biorhythm dysfunction, systematically described the potential underlying mechanisms, and provided some novel insights on the possible involvement of VAD in the development of some biorhythm-related diseases/disorders. VAD may affect the photo-response, sleep cycle, learning and memory, cell metabolism, and induce related disorders/diseases relevant to biological rhythm dysfunctions. Vitamin A and related metabolites may bind with their receptors (mainly RARs) to regulate gene expression (shown in the TTFLs), which may be the primary material basis underlying the biological rhythm dysfunctions induced by VAD. The possibility that RA signals may bridge the gap between VAD, circadian rhythm dysfunction, and related clinical disorders/diseases should be considered. However, although RA is the main active metabolite of vitamin A, and VAD is inevitably associated with abnormal RA signaling pathway, there is still a possibility that other factors that affect the functions of RA signaling pathways other than VAD may exist. Thus, the associations of RA signaling pathways with biological-rhythm disorders/diseases may not be exclusively due to VAD, and whether VAD induces related rhythm disorders/diseases *via* impaired RA signaling pathways warrants confirmation, especially using human studies with randomized controlled trials. More evidence of the influences of VAD on biological rhythms and its detailed mechanisms is warranted, which may be of great significance to clinical outcomes.

## Author contributions

XG: investigation and writing—original draft. HW: writing—original draft. JX: conceptualization, project administration, supervision, and writing—review and editing. HH: editing. All authors contributed to the article and approved the submitted version.

## Funding

This work was supported by the National Natural Science Foundation of China (81974486 and 81673189), Clinical Research Project of Shanghai Municipal Health Commission (202240392), Shanghai Jiao Tong University School of Medicine (20172016), and Shanghai Sailing Program (21YF1451500).

## Conflict of interest

The authors declare that the research was conducted in the absence of any commercial or financial relationships that could be construed as a potential conflict of interest.

## Publisher's note

All claims expressed in this article are solely those of the authors and do not necessarily represent those of their affiliated organizations, or those of the publisher, the editors and the reviewers. Any product that may be evaluated in this article, or claim that may be made by its manufacturer, is not guaranteed or endorsed by the publisher.

## Abbreviations

AANAT: arylalkylamine N-acetyltransferase
ARAT: acyl-CoA: retinol acyltransferase
ATRA: all-*trans* retinoic acid
BDNF: Brain-derived neurotrophic factor
BMAL1: brain and muscle Arnt-like protein 1
CLOCK: circadian locomotor output cycles kaput
CRF: corticotropin-releasing factor
CRY: cryptochrome
DHRS9: short-chain dehydrogenase/reductase (SDR family) member 9
HPA: hypothalamic-pituitary-adrenal axis
ipRGCs: intrinsically photosensitive retinal ganglion cells
IRBP: interphotoreceptor retinoid-binding protein
LRAT: lecithin:retinol acyltransferase
PER: Period
PVN: paraventricular nucleus
RA: retinoic acid
RALDH: retinaldehyde dehydrogenases
RARE: retinoic acid response elements
RARs: retinoic acid receptors
RBP: retinol-binding protein
RDHs: retinol dehydrogenases
REV-ERB: reverse erythroblastosis virus
RNRs: retinoid nuclear receptors
RORs: retinoid-related orphan receptors
RORE: ROR response element
RPE: retinal pigment epithelial
RXRs: retinoid X receptors
SCN: suprachiasmatic nucleus
SWS: slow wave sleep
TTFLs: transcription–translation feedback loops
VAD: vitamin A deficiency.
